# A Manual of Psychological Medicine

**Published:** 1858-10-01

**Authors:** 


*A Manual of Psychological Medicine.* By JOHN CHAS. BUCKNILL, M.D., and D. H. TUKE, M.D. 1858.

THE want of some systematic work to which the student and practitioner might refer for information on the varied questions involved in the diagnosis, treatment, and general management of insanity has so long been felt, that we hail with peculiar satisfaction the appearance of a "Manual of Psychological Medicine," by Drs. Bucknill and Tuke, both of whom are sufficiently known to the profession to warrant us in expecting much from their joint labours.

The work before us is divided into two portions, the first embracing the history, classification, statistics of insanity, is from the pen of Dr. Tuke; the remaining chapters, which treat of the diagnosis, pathology, and treatment of mental disorders, are by Dr. Bucknill.

Our space will not allow us to do more than briefly refer to some of the more important features of this excellent work.

The first two chapters are devoted to the history of insanity and its treatment as known to the ancients, in which the author displays an extensive acquaintance with the medical writings of antiquity, and a happy facility in selecting the leading features in these writings, which cannot fail to render this portion of the work interesting to those who regard an acquaintance with the progress of opinion as of importance to the psychological physician. These are followed by one on modern civilization in its bearings on insanity—a subject in itself almost inexhaustible, yet, nevertheless, of paramount importance, to the student of psychology, if he wish to render himself familiar with the influence which the ever-changing circumstances in which society is placed exercise in the production and modification of insanity. A true knowledge of the varied phenomena of insanity can only be obtained by patient and careful observation of man, his habits and peculiarities as he exists in general society, aided by the more limited but equally important observation of the various manifestations of disordered intellect as they are presented within the walls of an asylum. The alienist who limits his attention to the condition of the human mind as it manifests itself in hospitals for the insane can have but a very imperfect acquaintance with those nicer shades of mental disturbance by which sanity slides into insanity, and the moral and responsible man passes into the irresponsible maniac. To the physician engaged in the investigation of disorders of the mind, no subject which can, even in the remotest degree, exercise an influence on the social or moral condition of man, is without interest. The varied forms of mental disease, when viewed in large groups, have invariably been found to be modified by the prevailing social condition of the time, and we are satisfied that nothing tends so much to advance our knowledge of psychological medicine as the habit of taking a wide and comprehensive view of the various social conditions which, even in a remote degree, tend to develop insanity. This the author has done to the fullest extent in the present chapter, which is replete with important facts and valuable deductions. Dr. Tuke frankly contrasts the opposite conditions of barbarous and civilized life, and draws what seems to us a necessary conclusion, however repugnant it may be to our notions of modern civilization, in the following paragraph:—

"What can be a greater contrast than that which is presented by the untutored savage, on the one hand, and the member of a civilized community, on the other? The former passes his time chiefly in the open air, engaged in hunting and other pursuits, requiring much physical and but little mental exertion; never exhausts his brain by thinking, suffers very slightly from grief and sorrow, and knows little of the anxieties and sufferings connected with poverty. The latter, very generally, is obliged daily to infringe, more or less, the laws of health. He is subjected to the 'steady, soaking intoxication of habitual over-work.' If the brain demands rest, that rest is denied, and the brain perhaps goaded on by alcoholic stimulants. The very same person is possibly also the subject of ever-present anxiety and apprehension, in consequence of a precarious income. In a highly civilized community, the highest standard of intellectual attainment is constantly presented to the aspirations of its members; and minds, without reference to calibre, promiscuously enter the lists of an unequal contest. From these and other points of difference do we rashly draw the conclusion, that there are reasons for expecting a greater susceptibility to mental disorders among the civilized than the uncivilized nations of the world?"

Our limits will not allow us to consider this subject further; closely connected with which is that of the condition of the insane in modern times, on which the author bestows a chapter giving a succinct account of the change which has taken place in the management of the insane during the present century. On the subject of the classification of the different forms of mental disorder, Dr. Tuke, after a brief sketch of the various classifications of insanity adopted by different authors, submits the following modification, which, though imperfect, as all classifications must be until our knowledge of the pathology of insanity shall have been considerably extended, is perhaps, in the present state of our knowledge, as practically useful as any we could select, and conveys an approximative idea of the characters displayed by the leading types of mental disease.

"IDIOCY

| | |
|---|---|
| DEMENTIA . . . . | Primary.<br>Secondary. |
| DELUSIONAL INSANITY | Of a melancholy character.<br>Of an exalted character.<br>Of a destructive character. |
| EMOTIONAL INSANITY | Melancholia without delusions.<br>Mania with general extravagance of conduct ('moral insanity').<br>Mania with disposition to homicide.<br>Mania with disposition to suicide.<br>Mania with disposition to theft, &c., &c. |

MANIA . . . . . { Acute and Chronic.

PUERPERAL INSANITY.

"All of which may be complicated with epilepsy or general paralysis."

One hundred and sixty pages are devoted to the consideration of the various forms of mental disease enumerated above, the descriptions of which are forcible and accurate, displaying an extensive personal acquaintance with the subject, and amply illustrated by cases carefully selected from the practice of the author and from well-recognised authorities.

In considering this portion of the work, excellent as it is, we regret that Instinctive Mania has not received more attention and a fuller exposition of the grounds upon which it is recognised as a form of mental disease, believing as we do that a large proportion of cases generally, comprehended under the somewhat vague term emotional insanity, may be clearly traced to disorder of the instinctive faculties, which viewed through this light become capable of far more accurate definition than can possibly be whilst they are considered as but disordered emotions; indeed, the emotions themselves are closely allied to the instincts, and are to a considerable extent shared alike by man and animals.

Closely related as we are in physical organization to the other members of the animal kingdom, it is only reasonable to look for some similarity in our mental manifestations, and this, if it exist at all, is to be found in the instincts, which in the lower animals constitute the predominating, if not the sole guiding principle: in man these instinctive faculties are held in abeyance by the force of the will, any failing of which at once renders him a prey to their influence. Society presents us with numerous examples of men who, whilst in the possession of moderate intellectual powers, are, nevertheless, from disease or congenital defect, wanting in the power of self-control, and a prey to their instinctive propensities; a knowledge of which may be gained by a careful attention to their healthy and disordered manifestations in the lower animals. Comparative anatomy and physiology teach us much in reference to the structure and functions of the body; why may we not look to the same source for information in the higher attributes of our nature?

We have ventured upon these observations because we believe that the present state of our psychological knowledge warrants us in approaching much nearer to a correct estimate of what constitutes instinctive mania than has hitherto been done by writers in this department of medical science; and we feel that correct statement of what medical writers really include under this term is much needed to prevent so frequent collision between medical men and the opinions of the law.

In the chapter on the causes of insanity, Dr. Tuke enters at some length into the various questions of interest connected with this subject, and presents to us a valuable array of statistics in support of his opinions. Everything relating to the cause of insanity is involved in difficulty and doubt. Many of the supposed causes may be, and in numerous instances unquestionably are, but the first indications of mental decay. Vicious indulgences of every kind induce a proclivity to mental disease; but who can say how often these are but the first faint mutterings of the storm? The valuable work of Dr. B. A. Morel will lead us to look to those wide-spread social evils which result in the physical and moral degradation of a people, as a fertile source from whence both our criminal and insane population are augmented. Pauperism, and its attendant evils of filth and imperfect sanitary arrangements, play sad havoc with the mental powers of the lower orders, especially in large towns. The feeble parent begets a feeble offspring, who, born to all the miseries of poverty, sinks still lower in the scale of humanity; and thus pauperism becomes hereditary, and crime almost the only refuge for the man whose feeble mental and physical organization renders him unfit to compete in the battle of life with the healthy and robust. Thus through poverty do the causes of decay become cumulative, and our asylums become filled with unhappy imbeciles, the produce of our social condition. These questions perhaps belong to the political economist more than the medical man; yet they are of vital consequence to the alienist who seeks for the true cause of many of the diseases he has to treat. With this notice we must take leave of Dr. Tuke, to notice the important contributions to the work by his colleague.

Dr. Bucknill is so favourably known to the profession by his valuable contributions to psychological medicine, that it is scarcely necessary to do more, in noticing the portion of the work before us which he has contributed, than to say that it fully sustains the reputation he has already earned as a sound and comprehensive writer in everything pertaining to the insane.

The following paragraph from the chapter on the diagnosis of insanity, so fully indicates what we believe to be the qualities requisite for the investigation, that we cannot forbear quoting it as an example of the comprehensive view which our author takes of his subject:—

"The physician is compelled to bring to the investigation" (the diagnosis of insanity) "not only a knowledge of those functions which are subservient to the vegetative and animal life of the individual, but also a clear and analytical conception of those which collectively constitute mind. He must not only be a physician but a metaphysician; not, indeed, in the almost opprobrious sense of the term, but in that better sense which designates a lover of truth seeking to ascertain not the cause of mind or any other unattainable abstraction, but the laws of mind, which are as regular as any other natural laws, and the knowledge of which offers to philosophy a wholesome and legitimate object of research."

The whole chapter is admirably written, bearing the stamp of originality, and a perfect familiarity with the subject in all its bearings; free from vain speculations and hypotheses on the one hand, or a narrow dogmatism on the other. The maxims it contains may be followed as a safe and practical guide to the investigation of this subject, which forms perhaps the most anxious duty of the alienist. The author has paid considerable attention to the physiognomy of insanity. The varied appearances presented in the facial expression of the insane form one of the most important aids in the diagnosis of the disease, and to the skilful physician are frequently alone sufficient to guide his inquiries to the true nature of the malady. In the detection of feigned insanity, to which the concluding portion of the chapter is devoted, a knowledge of the facial expression of the different forms of mental disease is of much importance. Few men, unless possessed of extraordinary histrionic talent, and a familiarity with the insane, can at once and for a length of time copy not only the manners but also the expression of disease. In disease the expression of countenance is involuntary, requiring no effort of the will, whilst in the simulation this can only be sustained by the most constant effort; in feigned madness the patient is always anxious to convince bystanders of his insanity—an effort which, fortunately for psychology, few can sustain for any length of time. Dr. Bucknill has illustrated this subject with several important cases and much valuable criticism.

In the limits of a review it is impossible to notice even the leading features in the section devoted to the pathology of insanity; suffice it to say that throughout the author displays an acquaintance with all that is valuable in the writings of others on this obscure subject, and has brought to its investigation an extensive knowledge of the physiological science of the day. The following quotation will afford some idea of the grounds on which he endeavours to found a theory of mental pathology:—

"In default, therefore, of real knowledge respecting the conditions of nerve function, we must be satisfied with the recognition of the fact that the great organ of this function is subjected to the general laws of decay and reparation of animal tissues, and to some other laws having special reference to its own degeneration and repair."

"It is upon this physiological basis only that, in default of more precise and extensive knowledge of the changes in the nerve-cell and the generation of nerve-force, cerebral pathology can be established."

"The physiological principle upon which we have to build a system of cerebral pathology, is that mental health is dependent upon the due nutrition, stimulation, and repair of the brain; that is, upon the conditions of the exhaustion and reparation of its nerve substance being maintained in a healthy and regular state, and that mental disease results from the interruption or disturbance of these conditions."

Having thus stated the principles on which to establish his pathology, our author takes the following broad view of the organic conditions which lead to the production of insanity:—

"The brain, like every other organ of the body, for the perfect performance of its functions requires the perfect condition of its organization and its freedom from all pathological states whatever. Consequently, the existence of any pathological state in the organ of the mind will interrupt the functions of that organ, and produce a greater or less amount of disease of mind—that, is insanity."

How far the principles thus enunciated are sustained by the arguments of the author, we must leave his readers to judge; at all events, he has brought to their elucidation much sound reasoning, and a full appreciation of the importance of cerebral physiology in its bearings on the questions at issue.

The concluding chapter is occupied with the treatment of insanity, and embraces a full and comprehensive estimate of the relative value of the different medicinal remedies recommended by various writers.

The author speaks highly of the use of small doses of antimony in some forms of mania, not given with the view of producing nausea or depression. Of the value of this mode of treatment in the cases referred to by the author, we can bear the testimony of our own experience.

Opium, Dr. Bucknill, in common with most practitioners, regards as a valuable remedy in various forms of insanity, when selected with a due regard to their pathological condition.

The general or moral management of the insane recommended by the author is based upon the broad and liberal views entertained by most enlightened physicians of the present day. The insane are as amenable to all the good influences of a well-regulated household as any other portion of the community. Order, regularity, the judicious combination of firmness with kindness, and the force of example, are with the man of unsound mind, equally with the sane, the most powerful means of bringing him under control. A kind word and gentle persuasion will do more to calm the unruly than harsh and violent treatment.

To the physician and the philanthropist, the interior of an asylum having all the adjuncts of a comfortable house, and its inmates living together on terms of intimacy and kindness, must be a far more pleasing spectacle than the chains and bars of former days. In conclusion, we can confidently recommend this work to our readers as one of the best treatises on the subject in the English language. Throughout it bears the evidence of much careful study and accurate observation, which cannot fail to obtain for its authors an increased reputation.

